# The Prognostic Significance of *ZNF384* Fusions in Adult Ph-Negative B-Cell Precursor Acute Lymphoblastic Leukemia: A Comprehensive Cohort Study From a Single Chinese Center

**DOI:** 10.3389/fonc.2021.632532

**Published:** 2021-03-17

**Authors:** Ya-Zhen Qin, Qian Jiang, Lan-Ping Xu, Yu Wang, Hao Jiang, Feng-Ting Dao, Wen-Min Chen, Xiao-Su Zhao, Yan-Rong Liu, Xiao-Hui Zhang, Kai-Yan Liu, Xiao-Jun Huang

**Affiliations:** Peking University People’s Hospital, Peking University Institute of Hematology, Beijing Key Laboratory of Hematopoietic Stem Cell Transplantation, National Clinical Research Center for Hematologic Disease, Beijing, China

**Keywords:** B-cell precursor acute lymphoblastic leukemia, Ph-negative, adult, *ZNF384* fusions, relapse-free survival, B-other, allogeneic-hematological stem cell transplantation

## Abstract

Novel recurrent fusion gene types such as zinc finger protein 384 (*ZNF384*) fusions have been identified in B-cell precursor acute lymphoblastic leukemia (BCP-ALL) with the application of next-generation sequencing technologies. However, the comprehensive large-scale clinical cohort study for clarifying their prognostic significance remains scarce to date. A total of 242 consecutive adult Ph-negative BCP-ALL patients treated in our institute were retrospectively screened *ZNF384* fusions at diagnosis by multiplex real time quantitative PCR. *ZNF384* fusions were identified in 47 patients (19.4%) and all belonged to B-other ALL (having no high hyperdiploid karyotype, *BCR*-*ABL1*, *TCF3*-*PBX1*, *ETV6*-*RUNX1*, or *MLL* rearrangement). In the whole cohort, patients with *ZNF384* fusions had significantly higher 3-year relapse-free-survival (RFS) and tended to have a higher 3-year overall survival (OS) than those with no *ZNF384* fusions (80.1% *vs.* 52.5%, *P* = 0.013; 67.6% *vs.* 54.0%, *P* = 0.10). For patients receiving chemotherapy alone and received allogeneic-hematologic stem cell transplantation (allo-HSCT) were censored at the time of transplantation, patients with *ZNF384* fusions had both similar RFS and similar OS to B-other ALL patients with no *ZNF384* fusions (RFS: P =0.94 and 0.30; OS: P =0.94 and 0.51). For patients receiving transplantation, those with *ZNF384* fusions had significantly higher 3-year RFS than B-other ALL patients with no *ZNF384* fusions and their OS were similar (P = 0.022 and 0.24). Only two of 31 patients with *ZNF384* fusions and receiving allo-HSCT relapsed, individually occurred 66.8 and 69.8 months after transplantation. Therefore, *ZNF384* fusion is common in adult BCP-ALL, which may define a new group from BCP-ALL containing no classical fusion transcript with better prognosis through receiving allo-HSCT.

## Introduction

B-cell precursor acute lymphoblastic leukemia (BCP-ALL) is a molecular and cytogenetic heterogeneous disease. Oncogenic gene fusions induced by chromosomal rearrangements and specific aneuploidy patterns are the major hallmarks of BCP-ALL (1). In the past decade, knowledge about the genetic landscape of BCP-ALL has grown substantially with the application of next-generation sequencing technologies, especially transcriptome sequencing (2–11). In addition to the classical rearrangements such as *BCR*-*ABL1*, *TCF3*-*PBX1*, *ETV6*-*RUNX1*, and *MLL*, many novel recurrent fusion genes were identified, such as Ph-like fusions (2), *MEF2D* fusions (3), *DUX4* fusions (4, 7) and zinc finger protein 384 (*ZNF384*) fusions (6, 8).

Despite a high rate of response to induction chemotherapy, only 30%–40% of adult patients with ALL could achieve long-term remission. Thus, the aim of the identification of novel genomic abnormalities is to refine risk stratification to guide optimal treatment strategies and discover new therapies. However, the majority of published papers concerning the novel fusions of BCP-ALL focused on the identification of genomic lesions using new sequencing technologies, in which survival results were just showed briefly or not exhibited (2–11). As a result, the clinical cohort study which comprehensively investigates the characteristics and the prognosis of the individual novel fusions remains scarce to date.

The *ZNF384* gene is located at 12p13.3 and encodes a transcription factor that regulates promoters of the extracellular matrix genes (12). So far, eight fusion partners to *ZNF384* have been identified, and the reported frequencies of *ZNF384* fusions were varied in BCP-ALL. Similar to our preliminary transcriptome sequencing results (unpublished data), a report from Japanese Adult Leukemia Study Group (JALSG) showed that *ZNF384* fusions had the highest frequency among all novel fusions in adult Ph-negative BCP-ALL (9). In addition, the clinical significance of *ZNF384* fusions remains obscure; it was recognized as a good prognostic factor by some studies (7–9), whereas no significant impact on survival by others (5, 6, 13).

In the current study, by retrospectively screening *ZNF384* fusions in 242 consecutive adult Ph-negative BCP-ALL patients using multiplex real time quantitative polymerase chain reaction (RQ-PCR), we explored the incidence, characteristics and prognostic role of *ZNF384* fusions in BCP-ALL.

## Materials and Methods

### Patients and Treatment

A total of 242 adult Ph-negative BCP-ALL cases were included. They were consecutively diagnosed and received at least 1-course induction therapy in our institute from January, 2009 to August, 2018, and have available complementary DNA (cDNA) or RNA samples at diagnosis. One hundred sixteen were male and 126 were female patients, the median age at diagnosis was 32 years (range: 16–64 years). The patient diagnosis was based on bone marrow morphology, immunophenotyping, karyotyping, and molecular testing. The study was conducted in accordance with the Declaration of Helsinki and was approved by the Ethics Committee of the Peking University People’s Hospital. The informed consents were obtained from all subjects. The cutoff date for follow-up was February, 2020.

### Treatment

All patients received the same treatment protocols. As we reported previously, the chemotherapy procedure consisted of induction, consolidation, and maintenance chemotherapy (14). The CODP ± L regimen was used for induction. After achieving complete remission (CR) after induction, patients received the hyper-CVAD-based chemotherapy alone or chemotherapy followed by allogeneic-hematologic stem cell transplantation (allo-HSCT) (15). Chemotherapy was comprehensively described in our recently published paper (16). All patients were recommended to receive allo-HSCT after achieving CR1 unless the donor was absent, the performance status was poor or patient refused. The allo-HSCT indications, conditioning regimen, donor selection, graft-versus-host disease prophylaxis and the modified DLI regimen, were comprehensively described previously (17, 18).

### Multiplex RQ-PCR for the Detection of *ZNF384* fusions

Bone marrow samples collected at diagnosis were tested. Trizol Reagent (Invitrogen, CA, USA) was used to extract total RNA. A High Capacity cDNA Reverse Transcription Kit (Applied Biosystems, Foster City, CA, USA) was used to synthesize cDNA. The multiplex TaqMan-based RQ-PCR technology was used to measure the common five types of *ZNF384* related fusion transcript, *EP300*-*ZNF384*, *CREBBP*-*ZNF384*, *TCF3*-*ZNF384*, *EWSR1*-*ZNF384*, and *TAF15*-*ZNF384*. The primers and probes were designed according to the published and our unpublished transcriptome sequencing results using Primer Express software version 2.0. If the multiplex RQ-PCR showed exponential amplification, the split-out RQ-PCR with primer and probe sets for the individual fusion transcript was performed to identify partner.

### RQ-PCR for the Detection of Classical Fusion Transcript

*BCR*-*ABL1*, *TCF3*-*PBX1*, *ETV6*-*RUNX1*, and *MLL* rearrangement (*MLL*-*AF4*, *MLL*-*AF9*, *MLL*-*AF10*, *MLL*-*AF1*p, and *MLL*-*AF1*q) fusion transcripts were tested by RQ-PCR as described in our previous report (19). In addition, *IKZF1* deletion was tested in 146 patients who were diagnosed after 2014 by real-time PCR (20).

### Minimal Residual Disease (MRD) Monitoring

As we described previously, multiparameter flow cytometry was performed at diagnosis and used to monitoring MRD after treatment (16). The cutoff value of MRD level for the timepoint of at remission and after 1^st^ consolidation was set at 0.01%.

### Definitions and Statistical Analysis

Based on the MRC UKALLXII/ECOG E2993 adult ALL classification (21) and GRAALL-2003/2005 trial (22), low hypodiploidy or near triploidy, t(4;11), 14q32 translocation, and complex karyotype were classified as high-risk karyotypes, and high-risk was defined as having at least one of the following factors at diagnosis: age ≥35 years, WBC ≥30 x 10^9^/L, and high-risk karyotypes (23). The B-other group was defined as having no high hyperdiploid karyotype (51-65 chromosomes) and no *BCR*-*ABL1*, *TCF3*-*PBX1*, *ETV6*-*RUNX1*, or *MLL* rearrangement according to previous reports (24). CR means hematologic CR, which was defined as the presence of trilineage hematopoiesis and less than 5% BM blast cells, neutrophil counts of more than 1x10^9^/L, platelet counts of more than 100x10^9^/L, the absence of extramedullary disease and no recurrence for 4 weeks (25). Relapse-free survival (RFS) was measured from the date when CR was achieved and the event for it was relapse. The event for overall survival (OS) was death (regardless of the cause), and patients were queried at the date of last follow-up to determine whether they were still alive or censored on the date they were last known to be alive. The events for disease-free survival (DFS) included relapse and death. Pairwise comparisons of the variables between groups were performed using the Mann-Whitney U test for continuous variables and Fisher’s exact test for categorical variables. Survival functions were estimated using the Kaplan-Meier method and compared using the log-rank test. The variables with *P* < 0.20 by the univariate analysis were entered into a multivariate model using a Cox proportional hazards model to identify the most significant parameters associated with RFS and OS. The level for a statistically significant difference was set at *P* < 0.05. The SPSS 19 (IBM Corporation, Armonk, NY, USA) software package and GraphPad Prism 5 (GraphPad Software Inc., La Jolla, CA) were used for the data analysis.

## Results

### Patient Outcomes

The median follow-up time for the entire cohort was 19.5 months (range: 1.5–106.0 months). 150 (61.1%) patients were alive at the last follow-up with a median follow-up time of 36.0 months (range: 1.5–106.0 months). A total of 222 (91.7%) patients achieved CR after induction therapy, and 88 (39.6%) experienced a subsequent relapse with a median time to relapse of 7.2 months (range: 2.7–69.8 months). Of 222 patients achieved CR, 91 received chemotherapy alone, and 131 received chemotherapy followed by allo-HSCT (matched sibling donor, n = 37; haploidentical related donor, n = 91; matched unrelated donor, n = 3). Seven patients who relapsed after chemotherapy received salvage chemotherapy and received allotransplant after achieving CR2 (n = 6) or partial remission (PR, n = 1). The 3-year RFS, DFS and OS rates of the patients achieving CR were 58.0% (95% confidence interval (CI): 50.6%–64.7%), 52.3% (95% CI: 45.1%–59.0%) and 61.7% (95% CI: 54.2%–68.3%), respectively, and the 3-year OS rate of the entire cohort was 56.8% (95% CI: 49.6%–63.3%).

### Incidence of *ZNF384* Fusions

**Figure 1:**
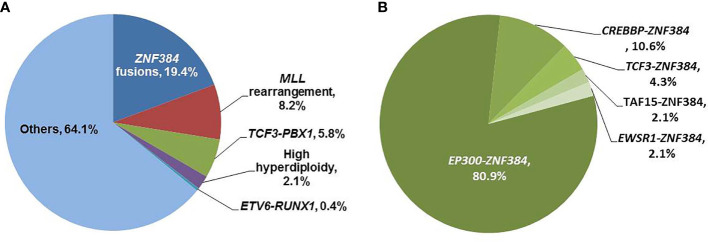
Distributions of *ZNF384* fusions. **(A)** Distributions of molecular and cytogenetic abnormalities in adult Ph-negative BCP-ALL patients. **(B)** Distributions of five types of fusions in patients with *ZNF384* fusions.

Of all 242 Ph-negative BCP-ALL patients, 47 patients (19.4%, **Figure 1A**) were identified *ZNF384* fusions: 38 had *EP300*-*ZNF384* (15.7%), five had *CREBBP*-*ZNF384* (2.1%), two had *TCF3*-*ZNF384* (0.82%), one had *TAF15*-*ZNF384* (0.41%), and one had *EWSR1*-*ZNF384* (0.41%). The most prevalent type, *EP300*-*ZNF384* accounted for 80.9% of all *ZNF384* fusions (**Figure 1B**).

In addition, 20 patients (8.3%) had a *MLL* rearrangement (19 *MLL*-*AF4* and 1 *MLL*-*AF1*p), 14 (5.8%) had the *TCF3*-*PBX1* fusion transcript, one (0.41%) had the *ETV6*-*RUNX1* fusion transcript, five (2.1%) had a high hyperdiploidy karyotype (**Figure 1A**). As a result, 202 (83.5%) patients belonged to the B-other ALL group. None of patients with *ZNF384* fusions had *MLL* rearrangement, *TCF3*-*PBX1* and *ETV6*-*RUNX1* fusion transcript and high hyperdiploidy karyotype. Therefore, all patients with *ZNF384* fusions belonged to B-other ALL, and the frequency of *ZNF384* fusions in B-other ALL was 23.3% (47/202).

The frequency of *ZNF384* fusions was significantly higher than *MLL* rearrangement, *TCF3*-*PBX1* and *ETV6*-*RUNX1* fusion transcript in Ph-negative BCP-ALL patients, respectively (*P* = 0.0005, < 0.0001, and < 0.0001).

### Characteristics of Patients With *ZNF384* Fusions

As shown in **Table 1**, in the whole cohort, *ZNF384* fusions were significantly related to higher platelet count at diagnosis (*P* < 0.0001), and tended to be related to non-complex karyotype (*P* = 0.081). Whereas, it had no relationship with age, sex, white blood cell (WBC) count, hemoglobin, *IKZF1* deletions and risk. Of 37 patients with *ZNF384* fusions and available karyotyping results, 27 (73.0%) had normal karyotype, one had complex karyotype, and none showed abnormality in 12p13.

**Table 1:** Relationship between *ZNF384* fusions and variables at diagnosis in adult Ph-negative BCP-ALL.

Variable	All	*ZNF384* fusions		*P* value
		Yes	No	
Number of patients	242	47	195	
Age (y, median, range)	32 (16*–*64)	28 (16*–*62)	33 (16*–*64)	0.56
Males (%)	116 (47.9%)	19 (40.4%)	97 (49.7%)	0.26
WBC count (×10^9^/L; median; range)	8.1 (0.3*–*52.2)	6.6 (1.6*–*225.0)	8.2 (0.3*–*512.2)	0.26
Hemoglobin (g/L)	87.0 (31.0*–*165.0)	95.0 (40.0*–*132.0)	84.0 (31.0*–*165.0)	0.12
Platelet count (×10^9^/L; median; range)	72.0 (0.6*–*510.0)	161.0 (12.0*–*368.0)	57.0 (0.6*–*510.0)	<0.0001
*IKZF1* deletion (%) (n = 138)	25 (18.8%)	3 (3/26, 11.5%)	22 (22/112, 19.6%)	0.41
Complex karyotype (%) (n = 184)	21 (11.4%)	1 (1/37, 2.7%)	20 (20/147, 13.6%)	0.081
High-risk (%) (n = 224)	140 (62.5%)	24 (24/45, 53.3%)	116 (116/179, 64.8%)	0.17

### Impact of *ZNF384* Fusions on CR Achievement

Totally 208 patients (86.0%) achieved CR after 1-course induction therapy, and the CR rate was similar between patients with *ZNF384* fusions and those with no fusions (43/47 *vs.* 165/195, 91.5% *vs.* 84.6%, *P* = 0.35).

### Impact of *ZNF384* Fusions on Survival in the Whole Cohort

**Figure 2:**
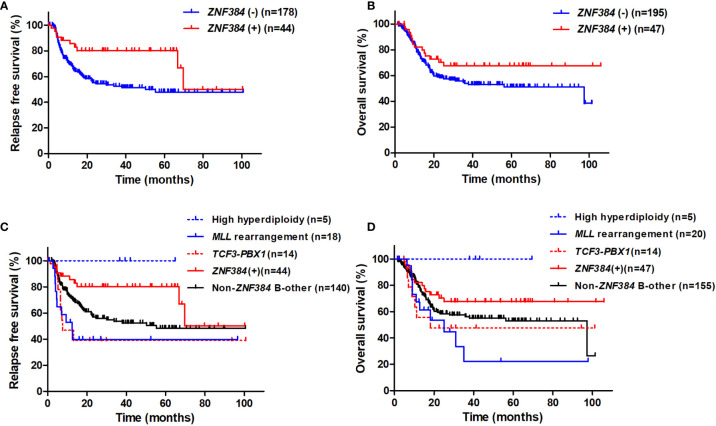
The impact of *ZNF384* fusions on RFS and OS in the whole cohort. **(A)** RFS, grouped by *ZNF384* fusions. **(B)** OS, grouped by *ZNF384* fusions. **(C)** RFS among subtypes. **(D)** OS among subtypes.

In the whole cohort, 222 patients achieved CR after induction therapy, and 44 of them had *ZNF384* fusions. *ZNF384* fusions was significantly related to a higher 3-year RFS rate (80.1% [95% CI: 64.0%–89.5%] *vs.* 52.5% [95% CI: 44.1%–60.2%], *P* = 0.011, **Figure 2A**), and there was a tendency that *ZNF384* fusions was related to a higher 3-year OS rate (67.6% [95% CI: 51.3%–79.5%] *vs.* 54.0% [95% CI: 45.9%–61.5%], *P* = 0.10, **Figure 2B**).

Survival among the subtypes was further compared. Patients with *ZNF384* fusions had significantly higher 3-year RFS rate than B-other ALL patients with no *ZNF384* fusions and those with *MLL* rearrangement and *TCF3*-*PBX1* fusion transcript, and had similar 3-year RFS rate to those with high hyperdiploidy karyotype, respectively (80.1% [95% CI: 64.0%–89.5%], 53.6% [95% CI: 44.1%–62.2%], 39.7% [95% CI: 17.0%–61.7%], 39.0% [95% CI: 14.4%–63.3%], and 100% [95% CI: 100.0%–100.0%], *P* = 0.021, 0.0039, 0.017, and 0.35, **Figure 2C**). Furthermore, patients with *ZNF384* fusions had significantly higher 3-year OS rate than those with *MLL* rearrangement, and had similar 3-year OS rate to B-other ALL patients with no *ZNF384* fusions, those with *TCF3*-*PBX1* fusion transcript and high hyperdiploidy karyotype, respectively (67.6% [95% CI: 51.4%–79.5%], 22.3% [95% CI: 4.0%–49.5%], 56.2% [95% CI: 47.2%–64.3%], 47.6% [95% CI: 20.2%–70.8%], 100% [95% CI: 100.0%–100.0%]; *P* = 0.024, 0.13, 0.13, and 0.21, **Figure 2D**).

Among patients with *ZNF384* fusions, both 3-year RFS rate and OS rate were similar between patients with *EP300*-*ZNF384* and those with others (RFS: 80.4% [95% CI: 61.3%–90.7%] *vs.* 77.8% [95% CI: 36.5%–94.0%], *P* = 0.76; OS: 62.3% [95% CI: 43.9%–76.2%] *vs.* 87.5% [95% CI: 38.7%–98.1%], *P* = 0.15).

### Impact of *ZNF384* Fusions on Survival Under Chemotherapy Treatment

Of patients receiving chemotherapy alone (n = 111, 91 achieved CR), *ZNF384* fusions had no significant impact on both 3-year RFS rate and OS rate (RFS: 28.1% [95% CI: 6.8%–54.9%] *vs.* 27.0% [95% CI: 16.5%–38.7%], P = 0.90, **Figure S1A**; OS: 25.0% [95% CI: 6.3%–49.9%] *vs.* 29.4% [95% CI: 18.9%–40.7%], *P* = 0.93, **Figure S1B**). Similar result existed if patients who received allo-HSCT were censored at the time of transplantation (RFS: 48.3% [95% CI: 16.8%–74.3%] *vs.* 35.5% [95% CI: 22.6%–48.6%], *P* = 0.18, **Figure S1C**; OS: 37.5% [95% CI: 12.2%–63.3%] *vs.* 31.7% [95% CI: 19.9%–44.2%], *P* = 0.42, **Figure S1D**).

**Figure 3:**
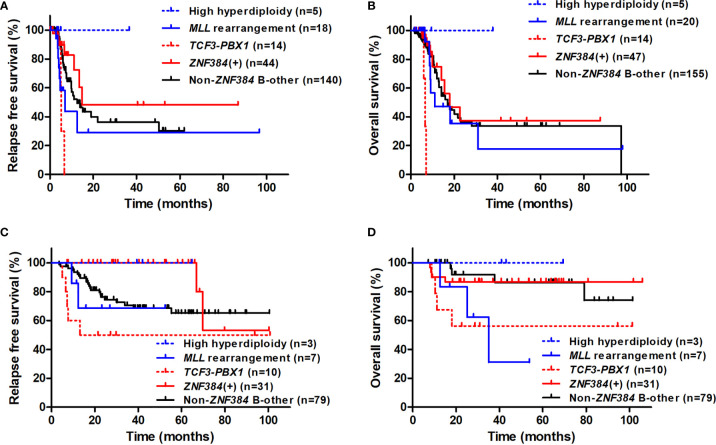
Comparison of RFS and OS among subtypes and considering treatment modality. **(A)** RFS of patients who received allo-HSCT were censored at the time of transplantation. **(B)** OS of patients who received allo-HSCT were censored at the time of transplantation. **(C)** RFS of patients receiving allo-HSCT. **(D)** OS of patients receiving allo-HSCT.

Comparisons among subtypes were performed (**Figures 3A, B**, **Figure S2**). For both patients receiving chemotherapy alone and, for all the patients, among whom received allo-HSCT were censored at the time of transplantation, patients with *ZNF384* fusions had both similar RFS rate and similar OS rate to B-other ALL patients with no *ZNF384* fusions (RFS: *P* = 0.94 and 0.30; OS: *P* = 0.94 and 0.51). Furthermore, for patients receiving chemotherapy alone, patients with *ZNF384* fusions had both similar RFS and similar OS to those with *MLL* rearrangement and *TCF3-PBX1* fusion transcript, respectively (RFS: *P* = 0.45 and 0.13; OS: *P* = 0.80 and 0.45). When patients who received allo-HSCT were censored at the time of transplantation, patients with *ZNF384* fusions had significantly higher 3-year RFS and OS than those with *TCF3-PBX1* fusion transcript (*P* = 0.0093 and 0.0009), and significantly higher 3-year RFS than and similar 3-year OS to those with *MLL* rearrangement (*P* = 0.036 and 0.80).

### Impact of *ZNF384* Fusions on Survival in Patients Receiving Allo-HSCT

Of 131 patients receiving allo-HSCT at CR1, those with *ZNF384* fusions had significantly higher 3-year RFS rate than patients with no *ZNF384* fusions (100% [95% CI: 100%–100%] *vs.* 70.2% [95% CI: 59.1%–78.8%], *P* = 0.017, **Figure S1E**), and there was a tendency that *ZNF384* fusions was related to a higher 3-year OS rate (86.9% [95% CI: 68.7%–94.9%] *vs.* 73.2% [95% CI: 62.4%–81.3%], *P* = 0.15, **Figure S1F**). Among 31 patients with *ZNF384* fusions and receiving allo-HSCT at CR1 (median follow-up 36.0 months [range: 2.0–102.0 months)], only two patients relapsed, individually occurred 66.8 and 69.8 months after transplantation.

Comparisons among subtypes were further performed (**Figures 3C, D**). For patients receiving transplantation, those with *ZNF384* fusions had significantly higher 3-year RFS than B-other ALL patients with no *ZNF384* fusions and their OS were similar (P = 0.022 and 0.24). Furthermore, patients with *ZNF384* fusions had significantly higher 3-year RFS and tended to have significant higher OS than those with *MLL* rearrangement and *TCF3-PBX1* fusion transcript, respectively (RFS: *P* = 0.0026 and 0.0038; OS: *P* = 0.062 and 0.065).

### Univariate and Multivariate Analysis

In the whole cohort, in addition to *ZNF384* fusion, Platelet count < 60×10^9^/L, high risk, treating with chemotherapy alone and not achieving CR within 4 weeks were significantly related to both lower RFS and lower OS (all *P* < 0.05, **Table 2**). The multivariate analysis showed that Platelet count<60×10^9^/L, not achieving CR within 4 weeks and treating with chemotherapy alone were independent poor prognostic factors for RFS, and not achieving CR within 4 weeks and treating with chemotherapy alone were independent poor prognostic factors for OS (**Table 3**). Similar results existed when analysis was performed in B-other ALL (**Tables S1, S2**). Therefore, *ZNF384* fusion was not an independent prognostic factor for both RFS and OS in both adult Ph-negative BCP-ALL and B-other patients.

**Table 2:** P value of univariate analysis in adult Ph-negative BCP-ALL.

Variable	RFS	OS
*ZNF384* fusions (Yes *vs.* No)	0.011	0.10
Sex (M *vs.* F)	0.32	0.30
Hemoglobin (g/L) (≤90 *vs.* >90)	0.95	0.45
Platelet count (×10^9^/L) (≥60 *vs.* <60)	0.0010	0.035
Risk (low *vs.* high) (n = 224)	0.024	0.0050
IKZF1 deletion (Yes *vs.* No) (n = 138)	0.50	0.3000
Treatment modality (allo-HSCT *vs.* chemotherapy alone)	<0.001	<0.001
Achieving CR within 4 weeks (Yes *vs.* No)	0.0070	<0.001
MRD>0.01% at remission (No *vs.* Yes) (n = 211)	0.97	0.40
MRD>0.01% after 1st consolidation (No *vs.* Yes) (n = 202)	0.65	0.75

**Table 3:** Multivariate analysis of RFS and OS in adult Ph-negative BCP-ALL.

Variable	RFS		OS	
	HR (95% CI)	*P* value	HR (95% CI)	*P* value
*ZNF384* fusions	–	0.48	–	0.57
Platelet count (×10^9^/L) (<60)	1.9 (1.2–2.9)	0.0065	–	0.41
Risk (n = 224)	–	0.39	–	0.17
Treatment modality (chemotherapy alone)	4.5 (2.8–7.1)	<0.001	6.3 (3.8–10.5)	<0.001
Achieving CR within 4 weeks (No)	6.3 (3.8–10.5)	0.0045	4.5 (2.8–7.1)	<0.001

## Discussion

Despite the identification of novel recurrent fusion genes in BCP-ALL with the application of next-generation sequencing technologies, the comprehensive large-scale clinical cohort study for clarifying their prognostic significance remains scarce to date. In the current study, by performing RQ-PCR to screen *ZNF384* fusions in 242 consecutive adult Ph-negative BCP-ALL cases at diagnosis, we found that the *ZNF384* fusions was related to higher RFS in the whole cohort. Analysis among subtypes implied that *ZNF384* fusions defined a new group from B-other ALL, which is related to higher RFS when receiving allo-HSCT but not receiving chemotherapy alone.

The *ZNF384* gene fusion was first discovered in acute leukemia with *EWSR1*-*ZNF384* and *TAF15*-*ZNF384* in 2002 (26). Because the corresponding chromosomal translocation is usually cryptic, the frequency of *ZNF384* fusions was underestimated, and all the relevant papers were case reports over the subsequent decade (27, 28). With the application of next-generation sequencing technologies, some novel recurrent genetic abnormalities in addition to *BCR*-*ABL1*, *ETV6*-*RUNX1*, *TCF3*-*PBX1*, and *MLL* rearrangement were identified. *ZNF384* fusion was found to be not rare in BCP-ALL, which was reported to comprise 2.0%–5.4% of pediatric BCP-ALL, 5% of pediatric B other-ALL and 5.7%–20.1% of adult Ph-negative BCP-ALL cases (5–9, 11, 13, 29). Similar to the Japanese report (9), *ZNF384* fusions was identified in 19.3% of our adult Ph-negative BCP-ALL patients, significantly more prevalent than classical fusions. Among all reports, the Australia group reported the lowest incidence in both pediatric and adult cases (8). It is unknown whether the prevalence of *ZNF384* fusions differs by race.

The prevalence of fusion partners reported was inconsistent among the transcriptome results. For samples from Tokyo Children’s Cancer Study Group (TCCSG) biobank, *TCF3* was the most prevalent *ZNF384* fusion partner (13). Whereas, Qian et al. found that among 231 pediatric BCP-ALL, *EP300* and *CREBBP* were the most prevalent partner of *ZNF384* with similar frequency (6). The Australia group reported that all AYA/adult pre-B-ALL patients had the same type, *EP300*-*ZNF384* (8). In a recent international study which delineated the transcriptome landscape of 1,223 BCP-ALL cases, *EP300*-*ZNF384* had the highest incidence within both pediatric and adult patients (10). In agreement with most of reports, we found that *EP300* was the predominant fusion partner which accounted for 80% of patients with *ZNF384* fusions. In addition, none of patients with *ZNF384* fusions were found chromosomal translocation involving 12p13. Therefore, *ZNF384* fusion was a common gene rearrangement in adult Ph-negative BCP-ALL which should be routinely screened at diagnosis using PCR technique.

The transcriptome studies which focusing on the identification of genomic abnormalities in BCP-ALL displayed discordant results on the prognosis of *ZNF384* fusions. A Chinese multicenter study which included 203 patients showed that there were no significant survival differences between patients with or without *ZNF384* fusions in either the adult or pediatric cohort (5). Qian et al. reported that in the context of the multi-institutional prospective Ma-Spore frontline ALL trial for children, *ZNF384* fusions had no significant effect on event-free survival (EFS) (6). In contrast, a report from Australia showed that the 8 *EP300*-*ZNF384* patients had favorable outcomes compared with other 85 BCP-ALL patients, and they explained that half of these patients were transplanted might be one reason (8). At the 2018 ASH meeting, Yasuda et al. reported that among 149 adult BCP-ALL patients treated with JALSG ALL202-O protocol, *ZNF384* fusions were associated with better DFS than B-others and should be classified as favorable-risk group (9). In the international 1223 delineating cases study (10), pediatric patients with *ZNF384* fusions were classified into low-risk group, and adult patients with *ZNF384* fusions were categorized into intermediate-risk group.

In our whole cohort, *ZNF384* fusion was related to higher RFS rate. Subgroup analysis showed that patients with *ZNF384* fusions had significantly higher RFS than B-other ALL with no *ZNF384* fusions, patients with *MLL* rearrangement and *TCF3*-*PBX1* fusions. We further showed that its prognosis was related to treatment modality; no impact on RFS under chemotherapy alone but was significantly related to higher RFS in patients receiving allo-HSCT when comparison was performed within B-other ALL. It implied that allo-HSCT patients might be the optimal treatment for adult BCP-ALL patients with *ZNF384* fusions, which is in accordance with the speculation of the Australia group (8). In addition, its non-significant impact on OS under both chemotherapy alone and HSCT and non-independent prognostic role in both B-other and Ph-negative BCP-ALL reflected that the prognostic significance of *ZNF384* fusion was not strong enough. Moreover, an interesting phenomenon is that only two patients with *ZNF384* fusions and receiving allo-HSCT relapsed and both relapsed over 5 years after transplantation. Recently, Nishimura et al. reported two cases of *TCF3*-*ZNF384*-positive pediatric ALL recurring more than 10 years after diagnosis (30). The underlying mechanism needs to be investigated to improve the long-term remission and survival.

Mechanism of *ZNF384* fusions has been investigated. *ZNF384* encodes a C2H2-type zinc finger protein with transcription activity. *BTLA*, *CLCF1*, and *GATA3* were individually demonstrated to be *ZNF384* target genes and transcription were upregulated by *ZNF384* fusions (6, 31). Qian et al. showed that *EP300*- and *CREBBP*-*ZNF384* fusions altered the potential of hematopoietic stem and progenitor cells (HSPCs) for lymphoid versus myeloid differentiation but did not improve the sustained growth of HSPCs in a serial replating experiment, which indicating that *ZNF384* fusions are not oncogenic by themselves but instead may increase the transforming potential of other oncogenic mutations (6). This might be in accordance with its moderate prognostic impact in ALL. The intermediate prognosis might be the reason that the outcomes of such patients were greatly improved by allo-HSCT. In addition, *EP300*- and *CREBBP*-*ZNF384* fusions resulted in loss of histone lysine acetyltransferase activity with concomitant global reduction of histone acetylation and increased sensitivity of leukemia cells to histone deacetylase inhibitors (6). This provides a clue for the use of targeting therapeutic agents in patients with *ZNF384* fusions.

Several limitations existed in the current study. First, this is a retrospective study, although the treatment regimens were uniform, dose reductions, discontinuations and treatment prolongations may occur during treatment for individuals. Furthermore, the chemotherapy for consolidation was not intensive enough. Second, IKZF1 deletion results were unavailable for a certain number of patients. Third, other gene mutations were not screened in the whole cohort.

In conclusion, *ZNF384* fusion is a common type of fusion gene which occurred in approximately one fifth of adult Ph negative BCP-ALL, which implied the necessity to screen it at diagnosis. *ZNF384* fusion is related to higher RFS in general. Furthermore, it may define a new group from BCP-ALL containing no classical fusion transcript with better prognosis through receiving allo-HSCT. The current results implied that allo-HSCT might be the optimal treatment for patients with *ZNF384* fusions. It needs to be validated by prospective multicenter trial in order to better stratify patients and improve the overall outcomes of adult BCP-ALL patients.

## Data Availability Statement

The raw data supporting the conclusions of this article will be made available by the authors, without undue reservation.

## Ethics Statement

The studies involving human participants were reviewed and approved by Peking University People’s Hospital. Written informed consent to participate in this study was provided by the participants’ legal guardian/next of kin.

## Author Contributions

Y-ZQ and QJ designed the research, Y-ZQ wrote the manuscript. Y-ZQ, F-TD, X-SZ and Y-RL analyzed the data. QJ, L-PX, YW, HJ, X-HZ, and K-YL collected the clinical data. X-JH revised the manuscript. All authors contributed to the article and approved the submitted version.

## Funding

This work was supported by the Capital’s Funds for Health Improvement and Research (2020-2Z-40811).

## Conflict of Interest

The authors declare that the research was conducted in the absence of any commercial or financial relationships that could be construed as a potential conflict of interest.
