# Supplementary material for: The Prognostic Significance of ZNF384 Fusions in Adult Ph-Negative B-Cell Precursor Acute Lymphoblastic Leukemia: A Comprehensive Cohort Study From a Single Chinese Center
Source: Front Oncol. 2021 Mar 17;11:632532. doi: 10.3389/fonc.2021.632532 (PMC8010301; doi:10.3389/fonc.2021.632532)
Supplement: Supplementary file 1 [file DataSheet_1.pdf]

Table S1. ***P*** **value of univariate analysis in B-other ALL patients**

Variable	RFS	OS
ZNF384 fusions (Yes vs No)	0.021	0.13
Sex (M vs F)	0.32	0.46
Hemoglobin (g/L) (≤ 90 vs > 90)	0.87	0.25
Platelet count ($\times 10^9$/L)(≥ 60 vs < 60)	0.0030	0.13
Risk (low vs high) (n=224)	0.060	0.023
IKZF1 deletion (Yes vs No)(n=116)	0.77	0.43
Treatment modality (allo-HSCT vs chemotherapy alone)	<0.001	<0.001
Achieving CR within 4 weeks (Yes vs No)	0.0010	<0.001
MRD>0.01% at remission (No vs Yes) (n=175)	0.6200	0.72
MRD>0.01% after 1st consolidation (No vs Yes) (n=167)	0.46	0.98

Table S2. Multivariate analysis of RFS and OS in B-other ALL patients

Variable	RFS		OS	
	HR (95%	*P* value	HR (95% CI)	*P* value
ZNF384 fusions (Yes vs No)	-	0.64	-	0.69
Platelet count (×109/L)(≥ 60 vs < 60)	1.9 (1.2-3.2）	0.011	-	0.68
Risk (low vs high)	-	0.49	-	0.39
Treatment modality (allo-HSCT vs chemotherapy alone)	6.6	<0.001	5.0 (3.0-8.5)	<0.001
Achieving CR within 4 weeks (Yes vs No)	3.8 (1.6-9.0)	0.0030	7.5 (4.1-13.4)	<0.001

Figure legends

Figure S1. The impact of ZNF384 fusions on RFS and OS considering treatment modality. (A) RFS of patients receiving chemotherapy alone. (B) OS of patients receiving chemotherapy alone. (C) RFS of patients who received allo-HSCT were censored at the time of transplantation. (D) OS of patients who received allo-HSCT were censored at the time of transplantation. (E) RFS of patients receiving allo-HSCT. (F) OS of patients receiving allo-HSCT.

Figure S2. Comparison of RFS and OS among subtypes in patients receiving chemotherapy alone. (A) RFS. (B) OS.
