# Supplementary figures and images for: The Prognostic Significance of ZNF384 Fusions in Adult Ph-Negative B-Cell Precursor Acute Lymphoblastic Leukemia: A Comprehensive Cohort Study From a Single Chinese Center
Source: Front Oncol. 2021 Mar 17;11:632532. doi: 10.3389/fonc.2021.632532 (PMC8010301; doi:10.3389/fonc.2021.632532)

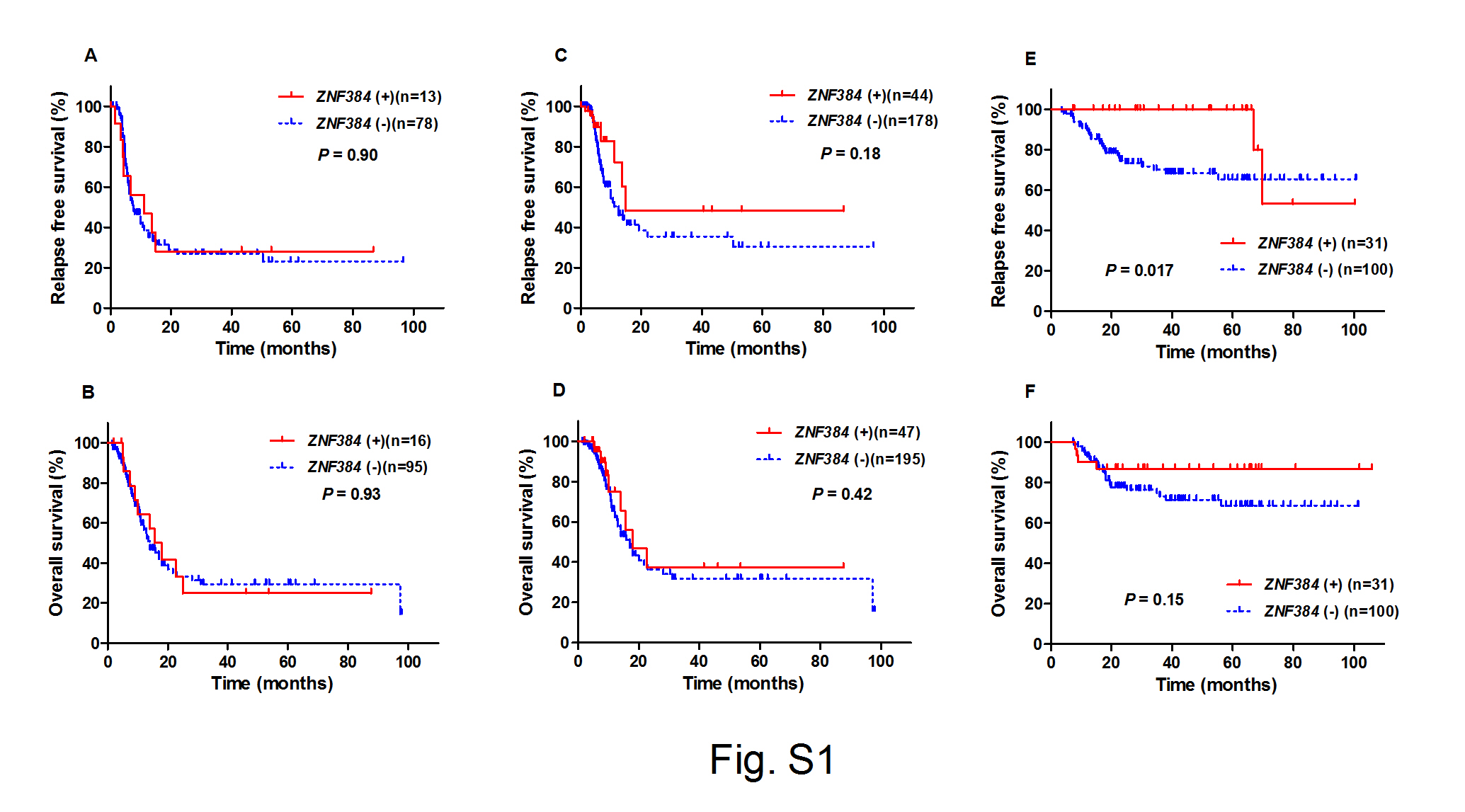

Supplement: Supplementary file 2 [file Image_1.jpeg]

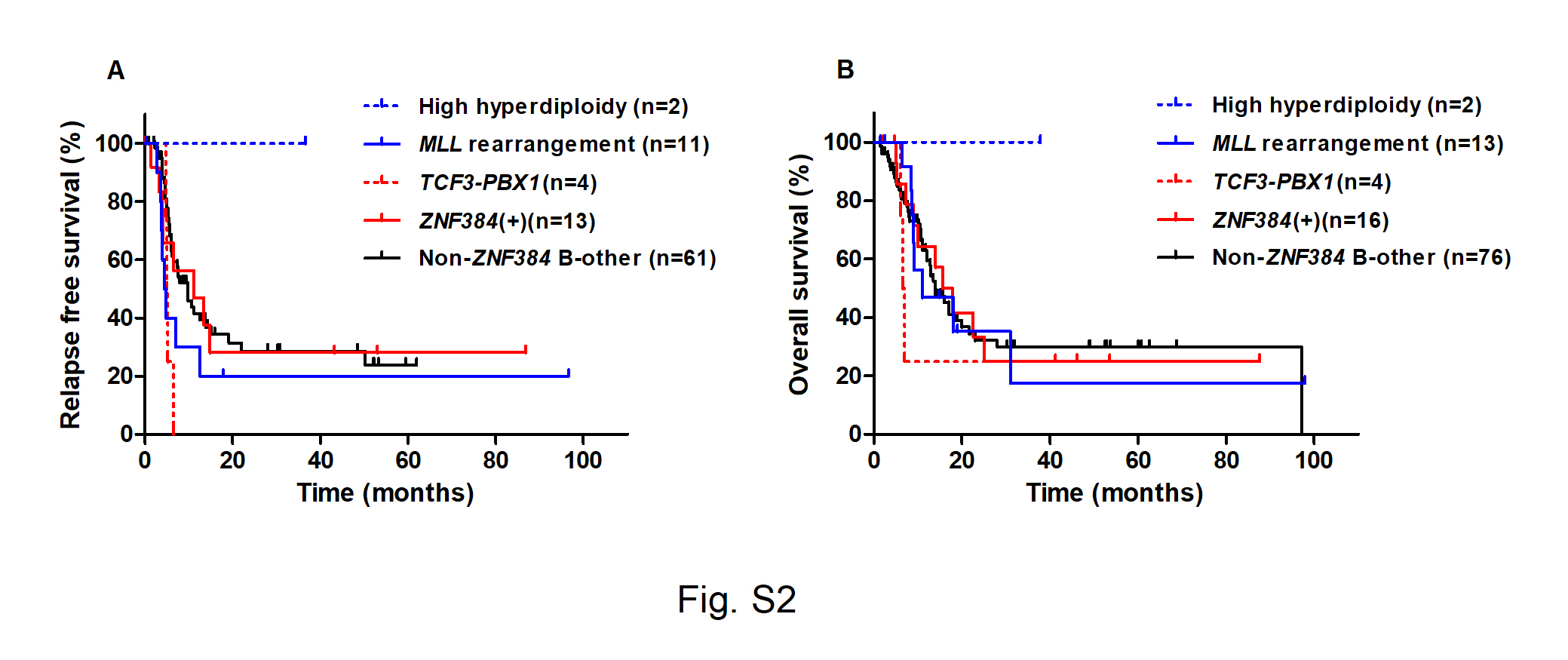

Supplement: Supplementary file 3 [file Image_2.jpg]
